# Incomplete spectrum QSM using support information

**DOI:** 10.3389/fnins.2023.1130524

**Published:** 2023-04-17

**Authors:** Patrick Fuchs, Karin Shmueli

**Affiliations:** Department of Medical Physics and Biomedical Engineering, University College London, London, United Kingdom

**Keywords:** QSM, compressed sensing, incomplete spectrum, dipole inversion, Fourier transform, regularization, magnetic susceptibility

## Abstract

**Introduction:**

Reconstructing a bounded object from incomplete k-space data is a well posed problem, and it was recently shown that this incomplete spectrum approach can be used to reconstruct undersampled MRI images with similar quality to compressed sensing approaches. Here, we apply this incomplete spectrum approach to the field-to-source inverse problem encountered in quantitative magnetic susceptibility mapping (QSM). The field-to-source problem is an ill-posed problem because of conical regions in frequency space where the dipole kernel is zero or very small, which leads to the kernel's inverse being ill-defined. These “ill-posed” regions typically lead to streaking artifacts in QSM reconstructions. In contrast to compressed sensing, our approach relies on knowledge of the image-space support, more commonly referred to as the mask, of our object as well as the region in k-space with ill-defined values. In the QSM case, this mask is usually available, as it is required for most QSM background field removal and reconstruction methods.

**Methods:**

We tuned the incomplete spectrum method (mask and band-limit) for QSM on a simulated dataset from the most recent QSM challenge and validated the QSM reconstruction results on brain images acquired in five healthy volunteers, comparing incomplete spectrum QSM to current state-of-the art-methods: FANSI, nonlinear dipole inversion, and conventional thresholded k-space division.

**Results:**

Without additional regularization, incomplete spectrum QSM performs slightly better than direct QSM reconstruction methods such as thresholded k-space division (PSNR of 39.9 vs. 39.4 of TKD on a simulated dataset) and provides susceptibility values in key iron-rich regions similar or slightly lower than state-of-the-art algorithms, but did not improve the PSNR in comparison to FANSI or nonlinear dipole inversion. With added (ℓ1-wavelet based) regularization the new approach produces results similar to compressed sensing based reconstructions (at sufficiently high levels of regularization).

**Discussion:**

Incomplete spectrum QSM provides a new approach to handle the “ill-posed” regions in the frequency-space data input to QSM.

## 1. Introduction

The magnetic susceptibility of tissue χ_*m*_ is related to perturbations in the magnetic field Δ*B*_0_ through convolution with the unit dipole field. These local field perturbations can be calculated from the phase variations measured in gradient-echo magnetic resonance imaging (MRI). In theory, reconstructing the underlying magnetic susceptibility from the local field perturbations requires deconvolution with the unit dipole field or dipole kernel. This is an ill-posed inverse problem because the dipole kernel contains zeroes on a conical surface in the frequency domain which lead to streaking artifacts in quantitative susceptibility mapping (QSM).

Over the years many different approaches to regularize this ill-posed problem have been proposed (Wang and Liu, 2015; Deistung et al., 2017; Shmueli, 2020): from direct approaches such as thresholding the dipole kernel (Shmueli et al., 2009; Schweser et al., 2013), to using iterative reconstruction methods (Wu et al., 2012; Kee et al., 2017; Milovic et al., 2018; Polak et al., 2020), and, most recently, deep-learning-based approaches (Bollmann et al., 2019; Jung et al., 2020, 2022).

Our approach to deal with this ill-posed region in frequency domain is to remove the affected data from the reconstruction. The QSM field-to-source inversion algorithm then needs to handle reconstructing the susceptibility from data that are incomplete in frequency domain (k-space: the MRI frequency domain). Reconstructing images from incomplete k-space data is often performed using compressed sensing (CS), where prior information on the sparsity of the image, in a (wavelet) transform domain, is used to aid reconstruction.

In contrast to CS, our approach does not rely on the incoherence of aliasing artifacts, but, rather, on a priori knowledge of the image-space support (more commonly referred to as the mask) of our object. Reconstructing a bounded object from incomplete k-space data is a well posed problem (Fuks, 1963; Papoulis, 1975), and it was recently shown that this incomplete spectrum (IS) approach can be used to reconstruct undersampled MRI images with similar quality to compressed sensing approaches, as shown by Rhebergen et al. (1997) and den Bouter et al. (2021).

In the case of QSM, the support information required for this incomplete spectruma approach is readily available as a binary mask as it is almost always used to remove background field contributions, and can generally be calculated from the magnitude images, see, for example, Smith (2002), Schweser et al. (2017), and Kiersnowski et al. (2022). No additional assumptions or priors are needed for the proposed approach. Our objective is to reconstruct a full magnetic susceptibility distribution (with a known mask) from incomplete k-space data. Here, we aimed to test this approach in QSM using both a numerical phantom and data acquired in five healthy volunteers. We investigated the effect of using different masks and band-limits (defining the incomplete region in k-space) on the reconstructed susceptibility maps using a numerical phantom. Using the volunteer data we compared our approach to conventional QSM reconstruction algorithms.

## 2. Theory: incomplete spectrum QSM reconstruction

We use the method first proposed in den Bouter et al. (2021) for MRI image reconstruction, which is a conjugate-gradient-least-squares (CGLS) algorithm applied to solve the normal equation of a space-limited and frequency-restricted Fourier transformation. In other words, instead of using the inverse fast Fourier transformation (FFT) to invert the Fourier transform equation


(1) $$\begin{array}{l} k=Fx,\; \end{array}$$

where *k* are the data in frequency domain, *x* are the data in image space and *F* is the (forward) Fourier transform, we limit the extent of *x* (through a mask, or support matrix *S*_*x*_ in image space) and restrict the frequency components of *k* (through a band limit or support matrix *S*_*k*_ in frequency domain). Then our space-limited and band-limited Fourier transform equation is


(2) $$\begin{array}{l} S_{k}k=S_{k}FS_{x}x.\; \end{array}$$

It is important to note that we choose the mask *S*_*x*_ such that *S*_*x*_*x* = *x*, or, in other words, so that the mask contains the support of the data *whose frequency domain we are attempting to reconstruct*. The normal equation of this model can then be solved for *x* using CGLS, as described in den Bouter et al. (2021).

In QSM, the local field perturbations, Δ*b*_0_ are related to the underlying magnetic susceptibility distribution χ_*m*_ through convolution with a dipole kernel *d*, first shown by Salomir et al. (2003) and Marques and Bowtell (2005)


(3) $$\begin{array}{l} \text{Δ}b_{0}=d*\chi_{m}.\; \end{array}$$

When transformed into frequency domain, this becomes an elementwise product according to the convolution theorem. This can be written as a matrix multiplication


(4) $$\begin{array}{l} b=F^{H}DF\chi ,\; \end{array}$$

where *F* is the forward Fourier transform matrix and *F*^*H*^(= *F*^−1^) is the inverse Fourier transform matrix, *D* is a diagonal matrix containing the Fourier transformed dipole kernel coefficients and *b* and χ are column vectors with the local field and magnetic susceptibility values, respectively.

One might think that it would be straightforward to compute the magnetic susceptibility by a simple deconvolution approach. However, this is, unfortunately, not possible as this inverse problem is not well posed because the dipole kernel *D* contains zeros on a conical surface in frequency domain. A simple approach to regularizing this problem is, for example, thresholding the kernel, i.e., replacing values in *D* that are too small with the signed threshold value, see Shmueli et al. (2009). Here, we use band limiting in the frequency domain to limit the frequency samples to include only the regions where the dipole kernel *D* has sufficiently large values, and the inverse is well-defined. Our initial equation for the susceptibility is


(5) $$\begin{array}{l} F\chi =D^{-1}Fb,\; \end{array}$$

where the susceptibility distribution χ is the source of the local magnetic field perturbations *b*. These are usually derived from the measured phase through multi-echo phase combination, phase unwrapping and background field removal, as described in Shmueli (2020), and as will be specified in the Section 3. Here, we space-limit (*S*_χ_) and band-limit (*S*_*k*_) Equation 5 as


(6) $$\begin{array}{l} S_{k}FS_{\chi }\chi =S_{k}\nu ,\; \end{array}$$

where ν = *D*^−1^*Fb* are our “input data” in the frequency domain. It is worth noting that the mask is strictly binary and a diagonal matrix in this formulation, which means *S*_χ_*S*_χ_ = *S*_χ_. In QSM this requirement is achieved through the background field removal step, where all field contributions from susceptibility sources outside of a pre-defined mask are removed from the (total) measured field perturbations (inside the mask). On the right-hand side of Equation 6 *S*_*k*_ excludes the ill-posed regions close to the cone at the magic angle. Therefore, we can relate our full discrete Fourier transform to the limited transform using


(7) $$\begin{array}{l} F\chi =FS_{\chi }\chi =S_{k}FS_{\chi }\chi +\left(I-S_{k}\right)FS_{\chi }\chi ,\; \end{array}$$

where *I* is the identity matrix, which leads to


(8) $$\begin{array}{l} F\chi =S_{k}\nu +\left(I-S_{k}\right)FS_{\chi }\chi .\; \end{array}$$

Taking the inverse Fourier transform *F*^*H*^ of both sides gives


(9) $$\begin{array}{l} \chi =F^{H}S_{k}\nu +F^{H}\left[\left(I-S_{k}\right)FS_{\chi }\chi \right].\; \end{array}$$

Left multiplying Equation 9 with the mask *S*_χ_, and transferring the second term to the left hand side gives


(10) $$\begin{array}{l} \left(I-S_{\chi }F^{H}\left[\left(I-S_{k}\right)FS_{\chi }\right]\right)S_{\chi }\chi =S_{\chi }F^{H}S_{k}\nu .\; \end{array}$$

It can be easily verified, as in den Bouter et al. (2021), that by defining the space-limited and band-limited discrete Fourier transform matrix *A* = *S*_*k*_*FS*_χ_, the above equation simplifies to the normal equation


(11) $$\begin{array}{l} A^{H}A\chi =A^{H}\nu .\; \end{array}$$

Which can then be solved in a least squares fashion, see, for example, Strang (2019), so using, for example, a CGLS algorithm.

### 2.1. Comparison to compressed sensing QSM reconstruction

In compressed sensing the optimization problem typically has a cost function of the form


(12) $$F_{CS}(\chi )={\left\|S_{k}\nu -S_{k}F\chi \right\|}_{2}+\text{λ}{\left\|\Psi \chi \right\|}_{1},$$

where Ψ is an appropriate sparsifying (often wavelet) transform, first described for MRI by Lustig et al. (2007).

As is well known, any solution that minimizes the least squares function


(13) $$F_{IS}(\chi )={\left\|S_{k}\nu -S_{k}FS_{\chi }\chi \right\|}_{2}$$

satisfies the normal Equation 11 as well. We can therefore use this form to compare the approaches. Here, we include the full expression for the space-limited and band-limited discrete Fourier transform matrix *A*(= *S*_*k*_*FS*_χ_) to illustrate its similarity with the compressed sensing framework. It should be noted that, though masking is often applied in iterative QSM reconstructions, this is the first time that the mask-based background field removal unique to QSM is leveraged to generate conditions through which the masking turns the problem into a well-posed integral equation.

To compare QSM reconstruction performance between the compressed sensing and incomplete spectrum approaches, we added the additional sparsity-promoting regularization term λ||Ψχ||_1_ to our incomplete spectrum optimization procedure (see Equation 12) in a “regularized incomplete spectrum” reconstruction. This was implemented in the Julia programming language using the “RegularizedLeastSquares” package, which is closely related to the MRI-specific work by Knopp and Grosser (2021). The sparsifying transform used in the “regularized incomplete spectrum approach” was a Daubechies wavelet with 2 vanishing moments (db2) (Vonesch et al., 2007), which is commonly used for this purpose (Majumdar and Ward, 2012).

## 3. Methods

### 3.1. Numerical phantom

To validate the incomplete spectrum QSM reconstruction method, we used a numerical phantom. This means that there was a known ground-truth susceptibility distribution so the QSM reconstruction error could be computed. Rather than using a reconstructed susceptibility map as a ground truth (which could lead to an “inverse crime” (Marques et al., 2021), we used the QSM reconstruction challenge 2.0 dataset from the QSM Challenge 2.0 Organization Committee et al. (2021) (see **Figure 2**), simulated using a comprehensive model.

The input to this method was the Sim2 dataset's local field map with signal to noise ratio SNR1. As the unwrapped, local field map was available, no additional pre-processing (i.e., background field removal) was necessary for susceptibility calculation. The brain mask used was the ground-truth mask provided with the dataset.

### 3.2. *In vivo* MRI acquisition

Since the simulated phantom is a high-resolution, high SNR data set, we also tested the performance of the incomplete spectrum method using brain images acquired *in vivo*. The *in vivo* dataset used is from Karsa et al. (2019), a gradient recalled echo acquisition at 3 Tesla with 1 mm isotropic resolution in five healthy volunteers. This was a 5 echo acquisition with echo times TE_1_ = 3ms, 5.4 ms echo spacing, 20° flip angle, TR = 29 ms, and pixel bandwidth = 270 Hz. Before susceptibility calculation, these data were processed according to the pipeline described in Karsa et al. (2019), described briefly here: the total field map was calculated from the multi-echo data using non-linear complex fitting (Liu et al., 2013). The total field map was then unwrapped using Laplacian unwrapping (Schweser et al., 2013), followed by background field removal with projection onto dipole fields (PDF) (Liu et al., 2011). The brain mask for background field removal was generated by combining a mask from the FMRIB Software Library's brain extraction tool (FSL BET) (Smith, 2002) (applied to the last-echo magnitude image) with a mask obtained by thresholding the inverse noise map derived from the non-linear fitting (Liu et al., 2013) as proposed by Karsa et al. (2020).

### 3.3. Choice of supports

The simulated numerical phantom dataset was used to investigate the effect of the choice of mask *S*_χ_ and band-limit *S*_*k*_ on the incomplete spectrum QSM reconstruction.

**Figure 1:**
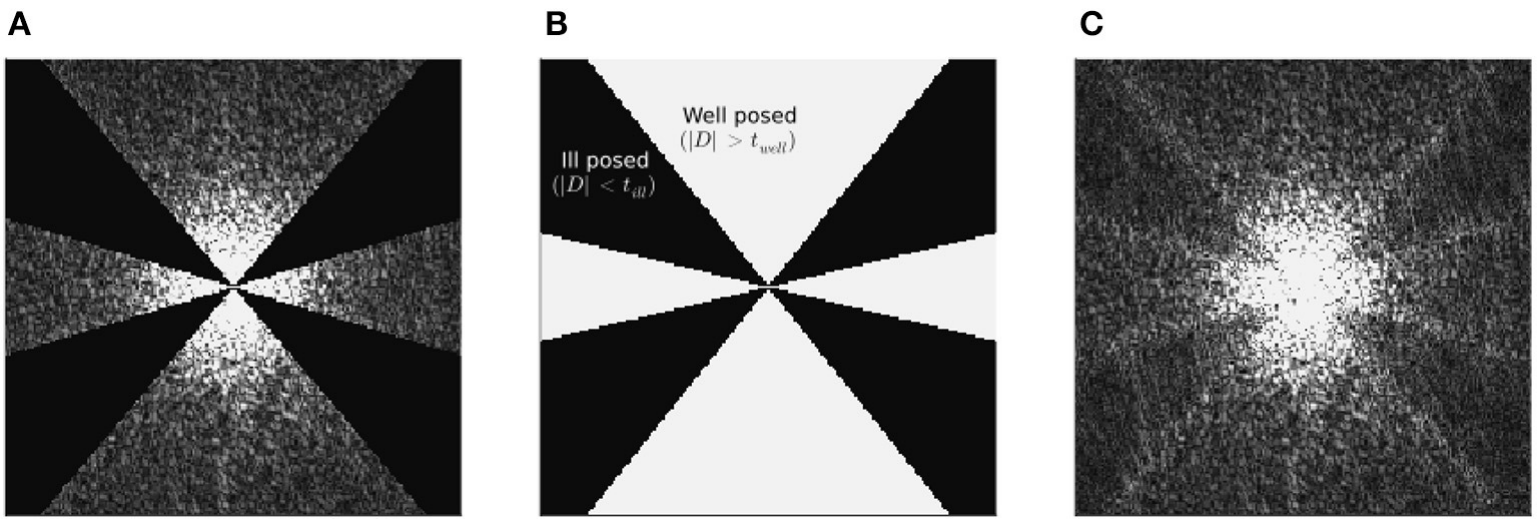
Challenge dataset ground truth frequency spectrum **(A)**, together with incomplete spectrum reconstructed k-space **(C)**, using the incomplete spectrum method with the (XSIM-optimal) band limit given in **(B)**. All data are absolute (e.g., |ν|) and slices are longitudinal (sagittal) in the frequency domain, and with the same grayscale (0…100 [a.u.]). The band limit boundaries, although much less stark than in **(A)**, are still visible in the reconstructed spectrum, which may explain the doubling of streaking artifacts around the calcificaiton in incomplete spectrum QSM reconstructions in **Figures 7**, **8**. The corresponding image space sagittal slice of **(C)** can be found in **Figure 3D**.

To illustrate the effect of the band limit on incomplete spectrum reconstruction, in Figure 1 we present a sagittal “slice” in k-space of: the k-space data (ν) input into conventional QSM algorithms, the band limit (*S*_*k*_), and the k-space of the incomplete spectrum QSM reconstruction (*Fχ*_*recon*_) corresponding to this band-limit. The reconstructed spectrum shows that the single streak in the input spectrum, which typically results in similar streaks in image space, is essentially split into two streaks at the boundaries of the discarded k-space, after being “filled-in” by the incomplete spectrum approach.

#### 3.3.1. Band-limit

As the purpose here was to choose a frequency domain support to exclude the regions where the QSM inverse problem is ill-posed, the frequency domain was divided into regions where the inverse problem is well-posed and ill-posed following the work of Schweser et al. (2012) and Wu et al. (2012). Fourier space was split into three regions according to the values of the dipole kernel. The well-posed region was defined as the kernel being larger than a threshold *t*_*well*_ or |*D*|>*t*_*well*_, and the ill-posed region was defined as |*D*| smaller than a threshold *t*_*ill*_ or |*D*| < *t*_*ill*_, where *t*_*ill*_ ≤ *t*_*well*_. We chose to limit the frequency domain to the well-posed regions, and do not consider a transition region between the thresholds for simplicity, therefore *t*_*ill*_ = *t*_*well*_. We investigated the relationship between QSM reconstruction quality and frequency domain support (in terms of choice of *t*_*well*_) using the simulated dataset. The image-space support was fixed to the challenge numerical phantom mask described above. The threshold value *t*_*well*_ was varied linearly from $\frac{1}{3}$ down to $\frac{1}{1000}$ in 100 steps. Note that we chose $\frac{1}{3}$ as the maximum threshold for the dipole kernel *D* because, although the absolute value of *D* has a maximum of $\frac{2}{3}$, using this as a maximum threshold value would result in a mask of all zeros which would exclude all the data from the inverse problem.

#### 3.3.2. Mask

As previously mentioned, for this incomplete spectrum approach to work, we require both an image space support (or mask) as well as a frequency domain support (or band-limit). As an image space support *S*_χ_, it is straightforward to use the mask that is conventionally used for background field removal in QSM (Schweser et al., 2017). In the case of brain imaging, which is a typical application of QSM, a brain mask can be readily calculated by thresholding one of the magnitude images or applying more sophisticated tools such as FSL BET (Smith, 2002) and noise-based thresholding (Karsa et al., 2020).

We investigated the effect of dilating and eroding the given binary mask by a few voxels to test the sensitivity of the incomplete spectrum QSM reconstruction to the mask *S*_χ_. In the numerical phantom simulation, we have perfect knowledge of the tissue boundaries and the support of our image-space susceptibility distribution. However, in real life the edges of this mask may not be perfectly determined. Therefore, using the numerical phantom, we explored both erosion as well as dilation of this support. The erosion and dilation were performed with a spherical kernel, ranging in diameter from 1 to 8 voxels. This led to an effective change in the support from −8 to +8 voxels. In this investigation the “optimal” band-limit as determined for the original brain mask (see below) was used. Note that in all cases, the input local field map (*b*) was masked using the same mask (*S*_χ_) as used by the incomplete spectrum algorithm otherwise the underlying assumption for this approach (i.e., *S*_χ_χ = χ) would be violated, and this mask was also used when computing the error metrics. We did not have to worry about incorporating erroneous information into the reconstruction as the local phase information is available throughout the domain, since there are no background fields in the simulation.

In the above investigations of the choice of supports (*S*_χ_ and *S*_*k*_) with the numerical phantom, we calculated both the susceptibility tuned XSIM metric from Milovic et al. (2019) used in the QSM challenge 2.0 (QSM Challenge 2.0 Organization Committee et al., 2021), as well as the peak signal to noise ratio (PSNR) given by Korhonen and You (2012). The XSIM metric is defined as


(14) $$\text{XSIM}(x,y)=\sum_{\text{ROI}}\frac{\left(2\mu_{x}\mu_{y}+K_{1})(2\sigma_{xy}+K_{2}\right)}{\left(\mu_{x}^{2}+\mu_{y}^{2}+K_{1}\right)\left(\sigma_{x}^{2}+\sigma_{y}^{2}+K_{2}\right)},$$

where μ_*i*_ is the window mean, and σ_*i*_ is the window variance (and covariance for σ_*ij*_). *K*_2_ and *K*_1_ are constants tuned to *K*_1_ = 0.01, *K*_2_ = 0.001 for susceptibility maps. To provide greater sensitivity to structural and local variance errors than other global metrics. We used this XSIM metric specifically because it has been shown to be more resistant to “metric hacking”, i.e. tuning hyperparameters to improve performance with respect to a specific image quality metric, as shown in Milovic et al. (2019) and QSM Challenge 2.0 Organization Committee et al. (2021).

### 3.4. QSM reconstruction comparison

The incomplete spectrum QSM reconstruction was applied *in vivo* with the threshold value optimized on the QSM Challenge dataset. In both datasets the novel incomplete spectrum method (as well as a compressed sensing regularized version of it) were compared to four different QSM reconstruction methods chosen from different categories of susceptibility calculation algorithms:

- Thresholded k-space division (TKD) (Shmueli et al., 2009), with point spread function correction for susceptibility underestimation as described by Schweser et al. (2013), was selected as a direct method.
- Non-linear total variation regularization (FANSI) (Milovic et al., 2018) and non-linear dipole inversion (NDI) (Polak et al., 2020) were selected as iterative methods with and without explicit regularization, respectively.
- A generic regularized least squares based compressed sensing reconstruction was used (Lustig et al., 2007).

In the numerical phantom the parameters of these reconstruction methods were tuned as follows, and the results can be found in Table 1.

**Table 1:** Reconstruction methods that were compared with the incomplete spectrum approach on the *in vivo* dataset and their parameters tuned for optimal PSNR in the numerical phantom.

**Method**	**Abbreviation**	**Parameters**	**XSIM**	**PSNR**
Thresholded k-space division	TKD	Threshold = $\frac{2}{3}$	0.88	39.4
Fast nonlinear susceptibility inversion	FANSI	$\lambda_{TV}=1\cdot 10^{-4}$, $\lambda_{TV}=1\cdot 10^{-5}$	0.92, **0.94**	**45.8**, 43.7
Nonlinear dipole inversion	NDI	Early stopping	0.90	40.2
Compressed sensing	CS	$\lambda_{\ell_{1}}=1\cdot 10^{-5}$, Ψ: Daubechies 2	0.89	40.6
Incomplete spectrum	IS	*t*_*well*_ = 0.25	0.88	39.9
Regularized IS	IS reg	*t*_*well*_ = 0.25, $\lambda_{\ell_{1}}=1\cdot 10^{-5}$, Ψ: Daubechies 2	0.89	39.7

Note that the regularized incomplete spectrum approach used the same parameters as the compressed sensing (CS) reconstruction. The XSIM and optimal PSNR in the numerical phantom are given for each reconstruction method. The bold values indicate the best performing algorithms for the respective metric.

#### 3.4.1. Parameter optimization for QSM reconstructions

For TKD the theoretical optimum threshold of $\frac{2}{3}$ was used. NDI uses automatic stopping, which requires no tuning. The compressed sensing and FANSI reconstructions were tuned in the numerical phantom using a parameter sweep to determine the regularization weights for optimal PSNR. For the CS reconstruction, the same band-limit (*S*_*k*_) as for the incomplete spectrum approach was used, and only the regularization weight was tuned (using a parameter sweep) for optimal PSNR. This same CS regularization weight was applied to regularize the incomplete spectrum method for ease of comparison.

#### 3.4.2. Region of interest comparison

**Figure 2:**
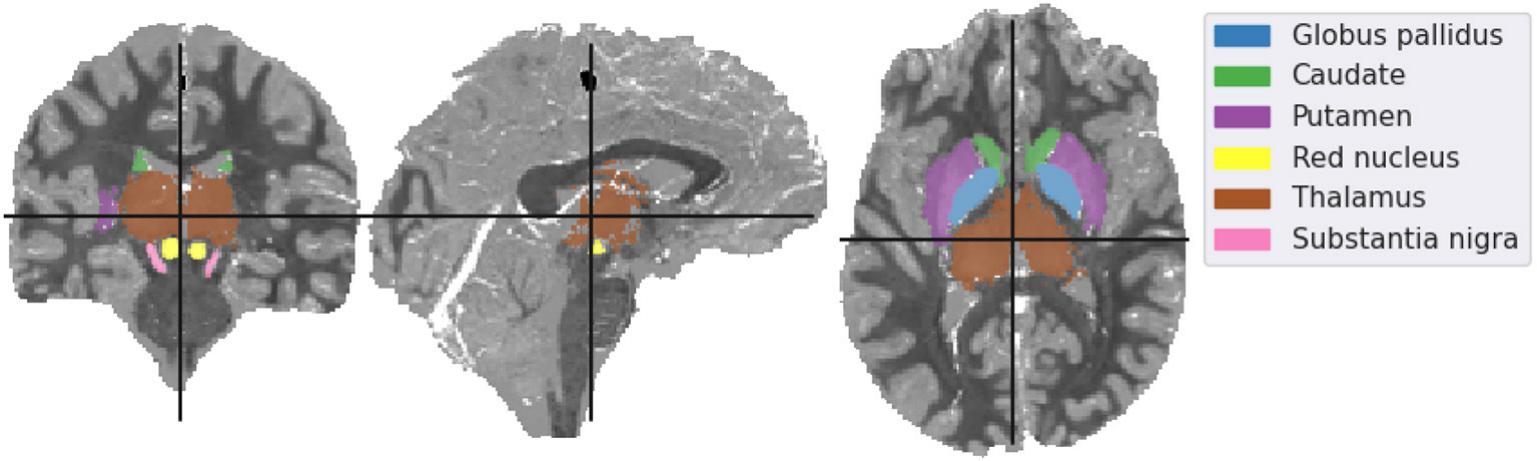
Mid-plane slices of the ground truth magnetic susceptibility distribution of the QSM challenge dataset (QSM Challenge 2.0 Organization Committee et al., 2021). ROIs used in the analysis are denoted overlaid on the midplane slices. The colormap of the susceptibility distribution is identical to that of the other presented reconstructions, i.e., between –0.1 and 0.1 ppm.

The mean susceptibility in the globus pallidus, caudate, putamen, red nucleus, thalamus, and substantia nigra were compared across all six different reconstruction methods (i.e. the incomplete spectrum approach, the regularized version, TKD, NDI, nlTV and compressed sensing) with the tuned regularization parameters in Table 1. Segmentations of these regions of interest (ROIs) were available for the simulated dataset (see Figure 2), and a segmentation performed using MRI cloud (Miller et al., 2014) was used for each of the volunteer datasets (see **Figure 9**). ROI mean susceptibility values were compared to the ground truth susceptibility in each ROI and averaged literature values from Bilgic et al. (2012) and Santin et al. (2017) for the numerical phantom and healthy volunteers, respectively.

## 4. Results

### 4.1. Choice of supports

**Figure 3:**
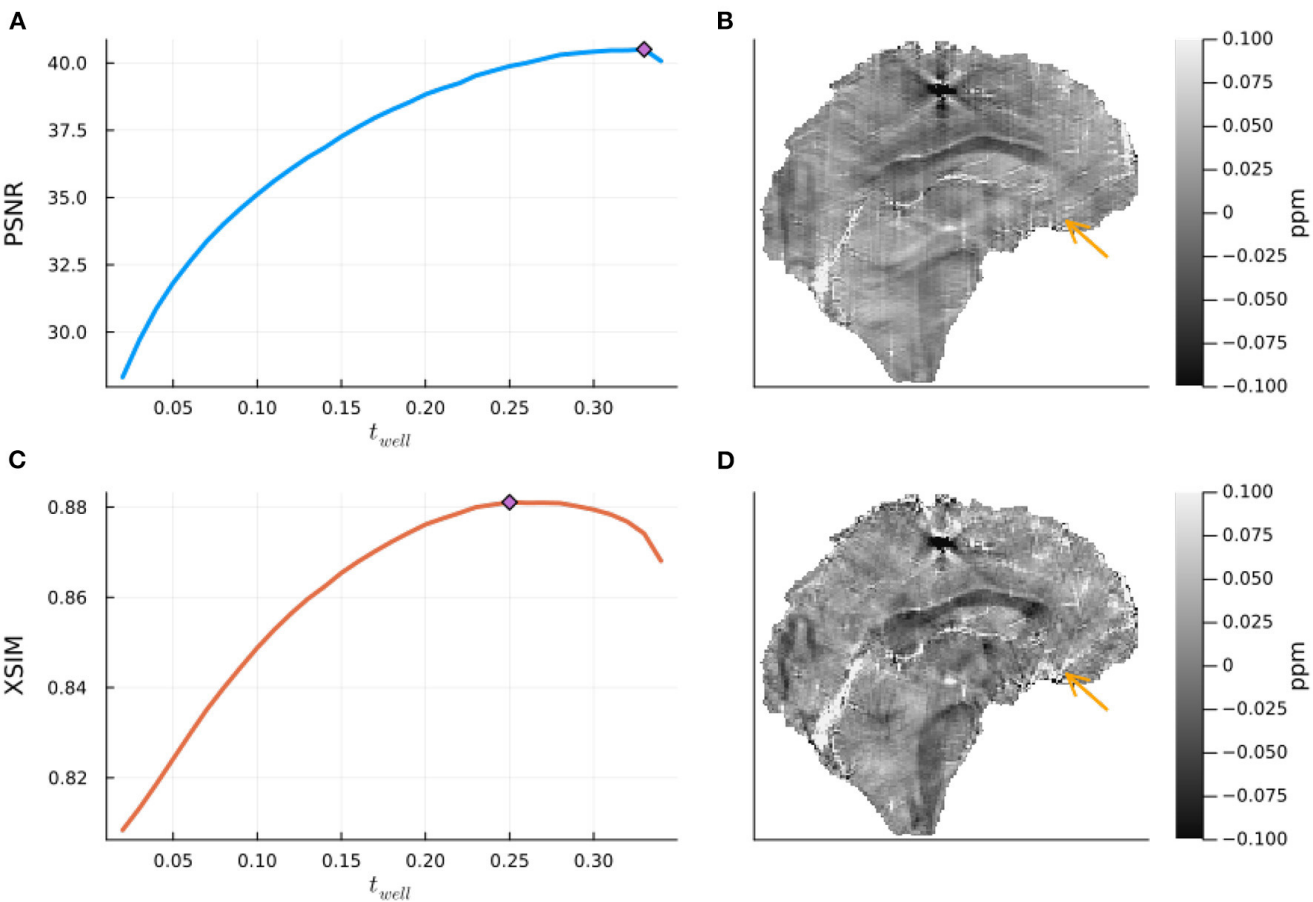
The effect of the band-limit *S*_*k*_ on the incomplete spectrum QSM reconstructed in the numerical phantom. On the left are the peak signal to noise Ratio (PSNR) and XSIM metrics for various threshold values *t*_well_, computed on the QSM challenge 2.0 dataset 2, with noise level 1 **(A, C)**. On the right are the corresponding optimal reconstructions (with the threshold for optimal PSNR (top) and XSIM (bottom) in a central sagittal slice **(B, D)**. The orange arrow points at a streaking artifact which is successfully suppressed at the higher regularization of the PSNR optimal value.

**Figure 4:**
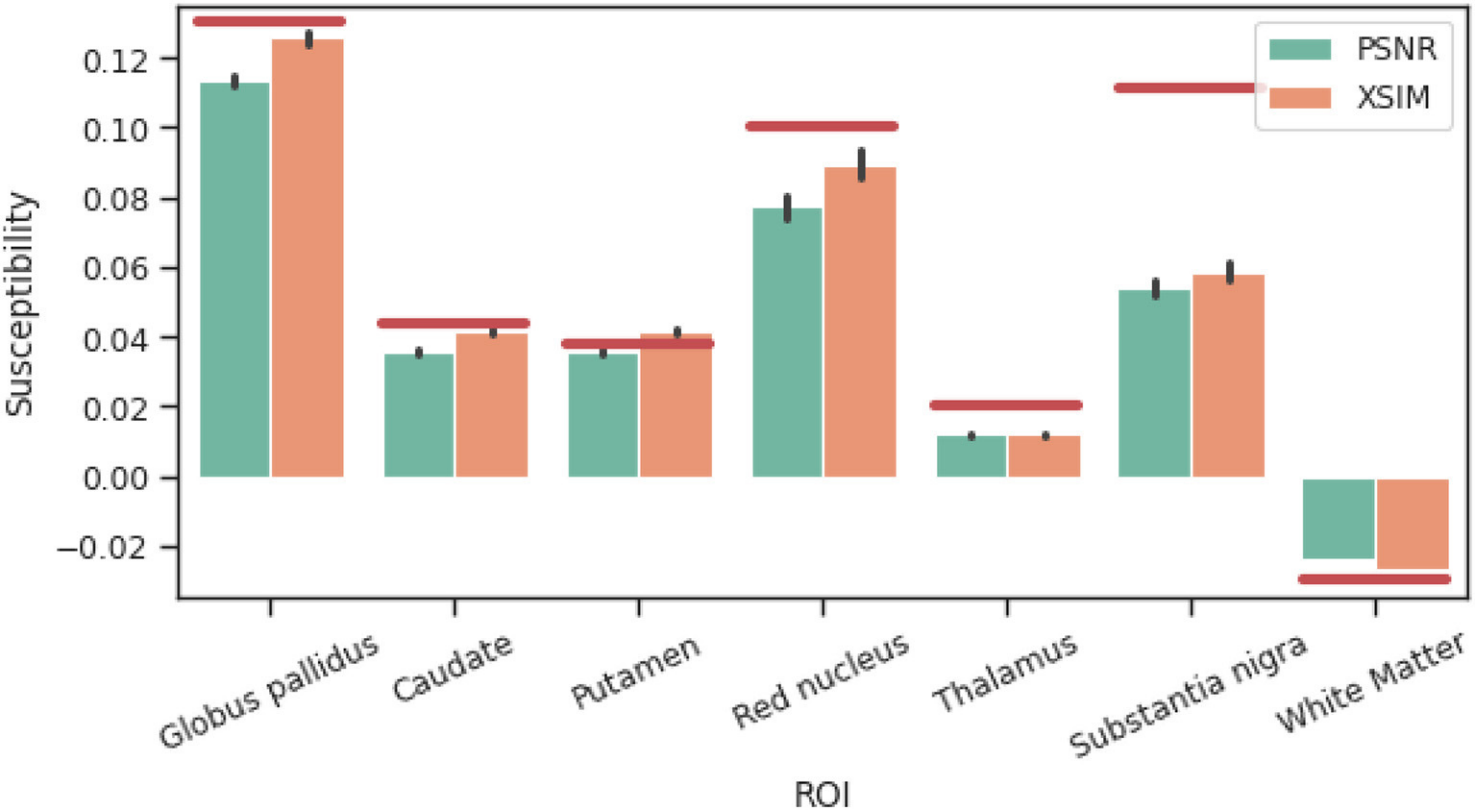
Comparison between XSIM-optimal and PSNR-optimal reconstructed susceptibility values (using the incomplete spectrum approach) for deep gray matter regions of interest in the challenge phantom. Red lines are ground truth susceptibility values, and vertical black lines signify 3 standard errors.

**Figure 5:**
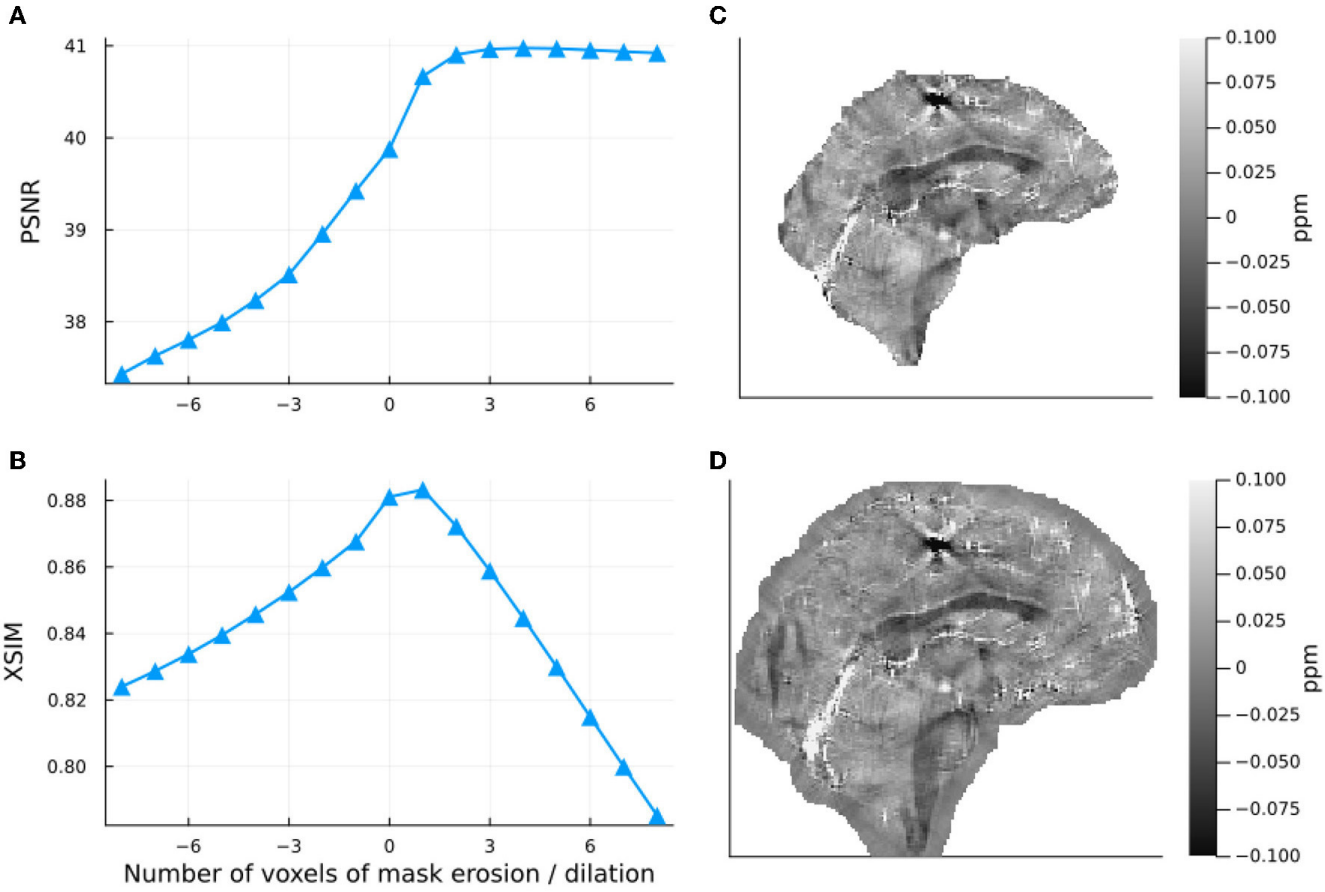
Effect of dilation and erosion of the brain mask or image support *S*_χ_ on the incomplete spectrum QSM reconstructed in the numerical phantom. Input data were masked with the same mask *S*_χ_ as that used in the reconstruction. On the left-hand side, PSNR **(A)** and XSIM **(B)** values are plotted for masks eroded and dilated by different numbers of voxels. The right-hand side shows incomplete spectrum QSM reconstructions with a mask eroded by 5 voxels **(C)** and a mask dilated by 5 voxels **(D)**.

The results of the band limit analysis can be found in Figures 3A, C with the sagittal slice of the PSNR-optimal and XSIM-optimal incomplete spectrum QSM reconstructions shown in Figures 3B, D for comparison. These show the effect on the reconstruction of changing the threshold *t*_well_, which changes the size of the well-posed k-space region. The PSNR-optimal reconstruction shows slightly less pronounced streaking artifacts than the XSIM-optimal reconstruction: See for example, the orange arrow in Figures 3B, D. However, the PSNR-optimal reconstruction is smoother and has much lower contrast than the XSIM-optimal reconstruction (Figures 3B, C). This loss in contrast is highlighted when comparing the two reconstructions in the six brain regions of interest, as shown in Figure 4. Based on these results, the XSIM-optimal threshold *t*_well_ = 0.25 was used for the reconstructions of the *in-vivo* datasets. The results of the space-limit *S*_χ_ investigation, where the effect of erosion and dilation of the mask are analyzed, are shown in Figure 5.

### 4.2. Parameter optimization for QSM reconstructions

The PSNR-optimal regularization weight for FANSI was 1·10^−4^ but this gave over-regularized reconstructions (i.e. smoothed and with loss of contrast) for the *in-vivo* dataset, and was therefore reduced to the XSIM-optimal weight of 1·10^−5^ which gave acceptable reconstructions with minimal streaking artifacts. Both regularization weights are included in Table 1 for reference. The regularization parameters giving optimal PSNR for each method in the numerical phantom are all shown in Table 1. These regularization parameters, tuned in the numerical phantom, were used to reconstruct all the *in-vivo* volunteer data.

### 4.3. QSM reconstruction comparison

The XSIM and PSNR metrics for all reconstruction methods on the challenge phantom data as well as their parameters can be found in Table 1. These metrics show that the incomplete spectrum approach is more accurate, with fewer streaking artifacts than the direct TKD approach. However, the incomplete spectrum approach performs slightly worse than the regularized iterative FANSI and CS methods. The performance of the algorithms are slightly different according to the XSIM and PSNR metrics. Adding regularization to the IS method offers minor improvement to XSIM at the cost of PSNR.

**Figure 6:**
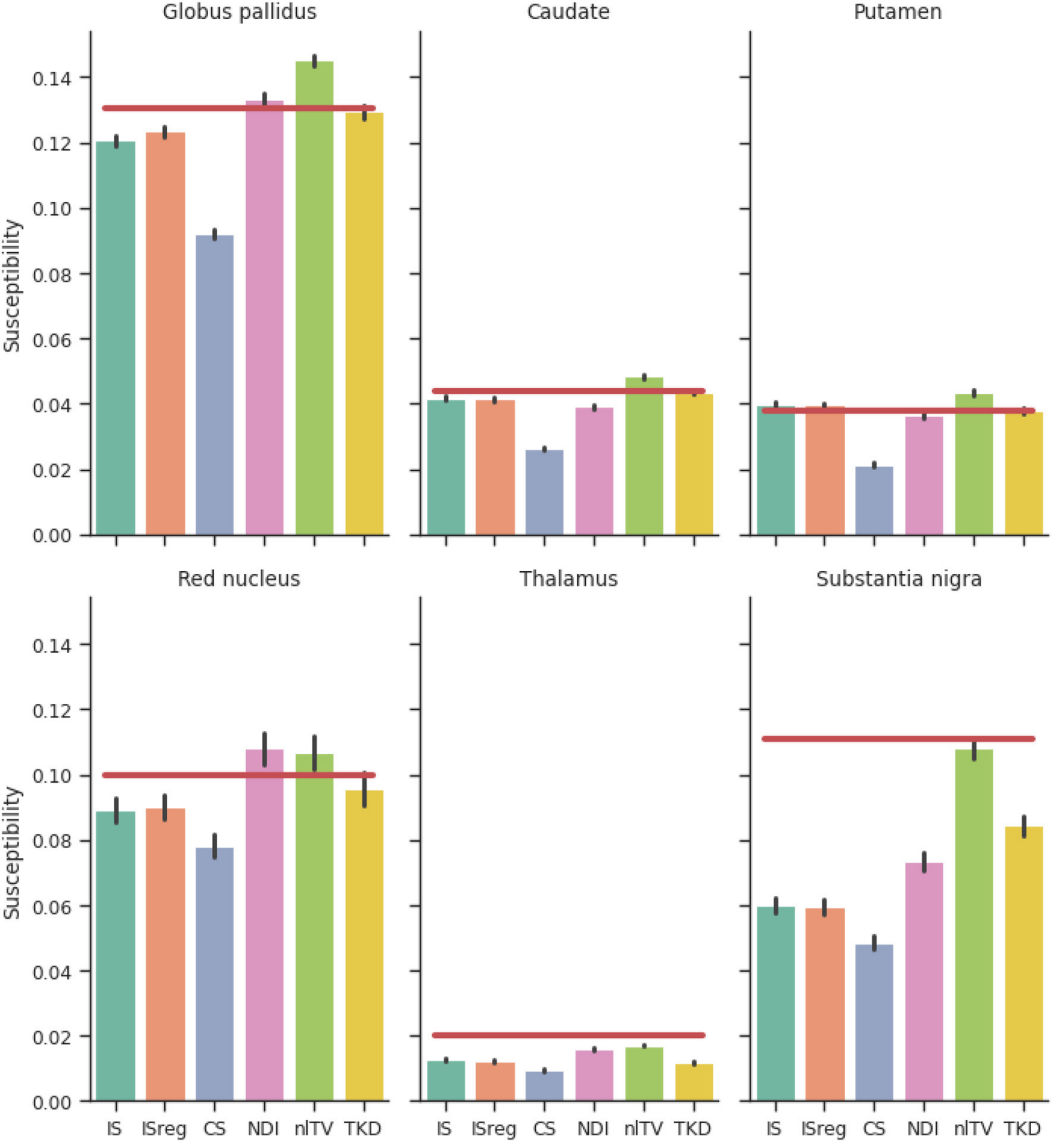
Reconstructed susceptibility values of the simulated dataset for deep gray matter regions of interest. Red lines are ground truth susceptibility values, and vertical black lines signify 3 standard errors. IS, Incomplete spectrum (*t*_well_ = 0.25); ISreg, Regularized IS (Same parameters as IS & CS); CS, Compressed sensing (Ψ: Daubechies 2, $\lambda_{\ell_{1}}=1\cdot 10^{-5}$); NDI, Nonlinear Dipole Inversion (w. automatic stopping); FANSI, Fast Nonlinear Susceptibility Inversion ($\lambda_{TV}=1\cdot 10^{-5}$); TKD, Thresholded k-space division (λ = 2/3; w. PSF correction).

Figure 6 shows a comparison of ROI mean susceptibility values in the simulated phantom for the different QSM reconstruction methods. For reference, ground truth ROI susceptibility values are given by the horizontal red lines.

**Figure 7:**
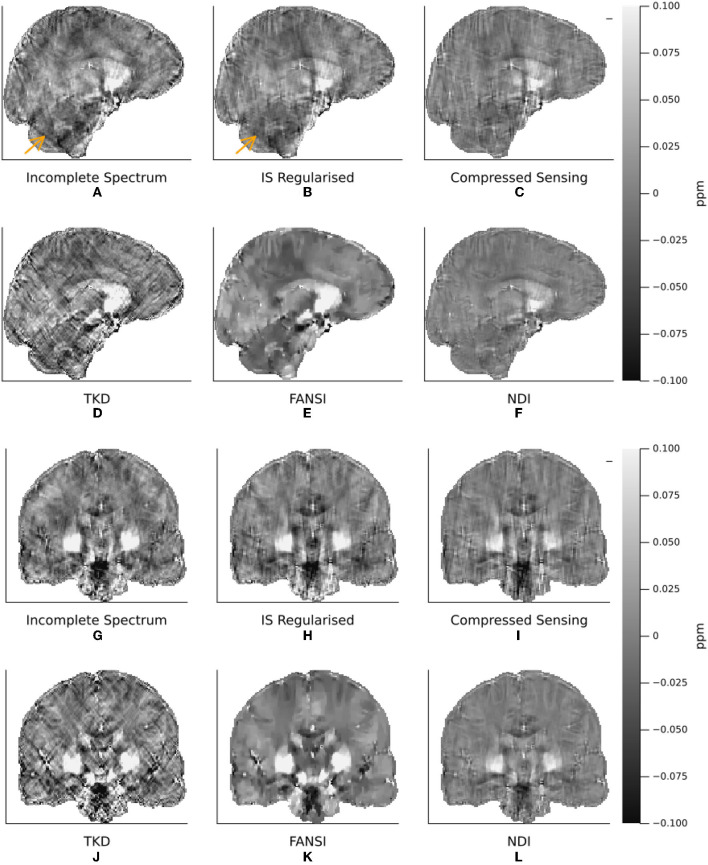
A Comparison of Incomplete Spectrum QSM with Conventional QSM Reconstruction Methods in a representative healthy volunteer. Coronal **(A–F)** and sagittal **(G–L)** and slices are shown to highlight streaking artifacts. The incomplete spectrum reconstruction **(A, G)** used the XSIM-optimal regularization weight determined from the challenge dataset, (*t*_*well*_ = 0.25). PSNR/XSIM optimal regularization parameters for the other QSM methods are given in Table 1. The reconstructions are ordered as incomplete spectrum **(A, G)**, regularized incomplete spectrum **(B, H)**, compressed sensing **(C, I)** (top row). Followed by TKD **(D, J)**, FANSI **(E, K)**, and NDI **(F, L)**. The orange arrow highlights a streaking artifact that is reduced by the added regularization in the regularized incomplete spectrum reconstruction **(B)**.

**Figure 8:**
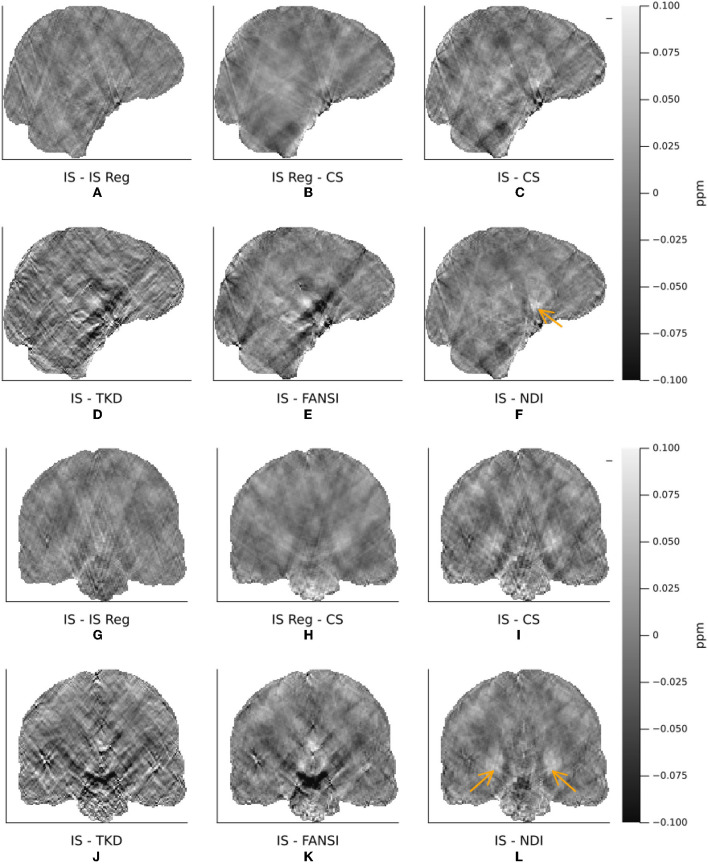
Differences between incomplete spectrum and conventional QSM reconstructions in the same representative healthy volunteer as shown in Figure 7. The reconstructions are ordered as incomplete spectrum **(A, G)**, regularized incomplete spectrum **(B, H)**, compressed sensing **(C, I)** (top row). Followed by TKD **(D, J)**, FANSI **(E, K)**, and NDI **(F, L)**.

The XSIM-optimal threshold of *t*_*well*_ = 0.25 was used in the *in vivo* reconstructions, as it provided higher contrast and less smooth susceptibility maps than the PSNR optimal threshold. All six QSM reconstruction methods are visually compared in Figure 7, where we have displayed sagittal and coronal slices to emphasize QSM contrast between brain structures typically of interest as well as the level of streaking artifacts. Figure 8 shows difference maps between our proposed incomplete spectrum QSM reconstruction method and the conventional QSM reconstruction methods investigated. The difference maps show that most of the differences between the conventional QSM reconstructions and the incomplete spectrum reconstruction seem to be residual streaking susceptibility differences and not anatomical artifacts although some deep-brain gray-matter regions appear brighter in the IS QSM, particularly compared with NDI, highlighted by orange arrows in Figures 8F, L.

**Figure 9:**
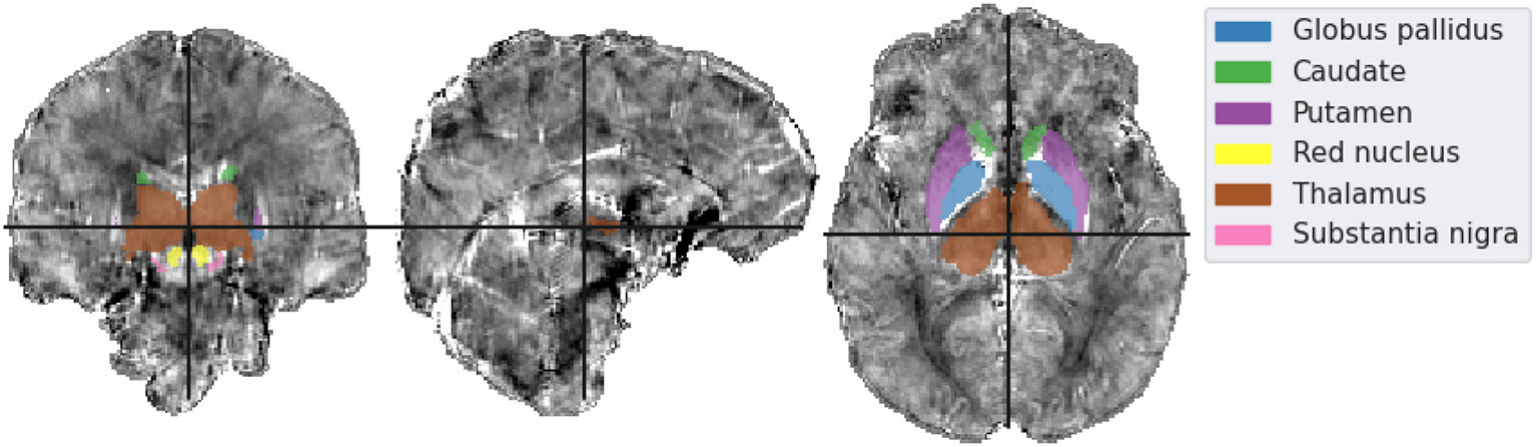
Mid-plane slices of the incomplete spectrum reconstruction of the same representative healthy volunteer presented in Figures 7, 8. ROIs segmented using MRI Cloud and used in the analysis are overlaid. The colormap of the susceptibility distribution is identical to that of the other presented reconstructions, i.e., between –0.1 and 0.1 ppm.

**Figure 10:**
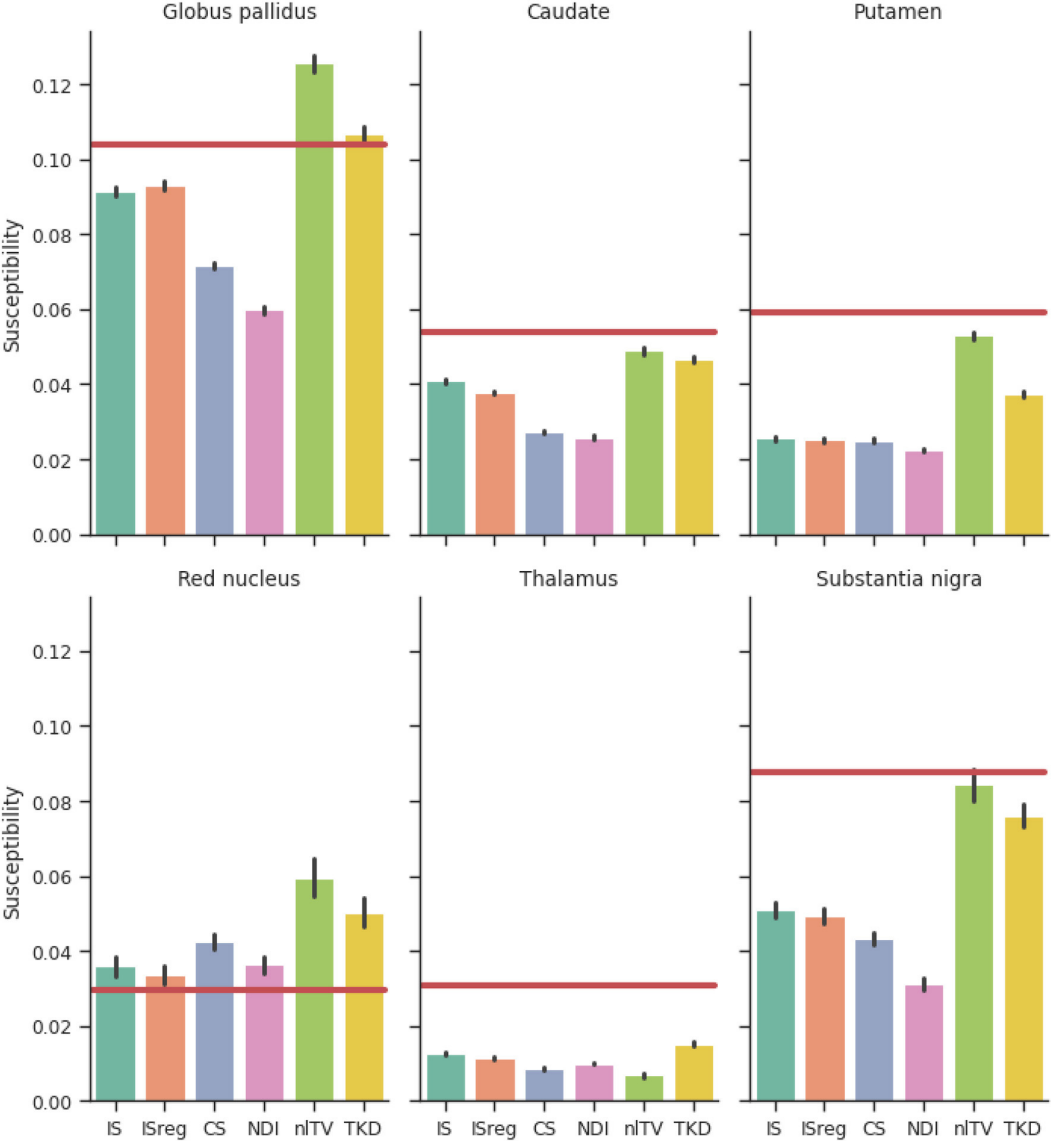
Reconstructed susceptibility values for deep gray matter regions of interest in the 5 volunteer datasets. Red lines are averaged literature values from Bilgic et al. (2012) and Santin et al. (2017), and vertical black lines signify 3 standard errors. IS, Incomplete spectrum (*t*_well_ = 0.25); ISreg, Regularized IS (Same parameters as IS & CS); CS, Compressed sensing (Ψ: Daubechies 2, $\lambda_{\ell_{1}}=1\cdot 10^{-5}$); NDI, Nonlinear Dipole Inversion (w. automatic stopping); FANSI, Fast Nonlinear Susceptibility Inversion ($\lambda_{TV}=1\cdot 10^{-5}$); TKD, Thresholded k-space division (λ = 2/3, w. PSF correction).

In Figure 9 the ROI segmentation of a representative healthy volunteer is shown. And Figure 10 shows a comparison of ROI mean susceptibility values, averaged over all five volunteers, for the different QSM reconstruction methods. For reference, literature ROI mean susceptibility values are given by the horizontal red lines.

## 5. Discussion and conclusions

Here, we demonstrated a new incomplete spectrum QSM reconstruction approach based on excluding “ill-posed” regions from the frequency domain. Without additional regularization, incomplete spectrum QSM reconstruction showed lower levels of streaking artifacts compared to direct QSM reconstruction and similar accuracy to state-of-the art QSM reconstruction algorithms.

### 5.1. Numerical phantom

Investigations in the numerical phantom from the QSM challenge showed that there is a frequency-space band limit *S*_*k*_ that is optimal for this dataset Figure 3, which is relatively robust (a 50% reduction of the threshold resulted in a 7.1 % decrease in the PSNR metric, and decreased the XSIM metric by 2.8%). This XSIM optimal threshold value of *t*_*well*_ = 0.25 seems high, as it fills in almost two thirds of k-space. The results, however, do not indicate over-regularization (through the loss of anatomical contrast or smoothing), and the incomplete spectrum method provided high-quality reconstructions *in vivo* without requiring additional tuning, further supporting its robustness.

Masking, although non-trivial (Smith, 2002), is already an integral part of most QSM pipelines i.e., for background field removal (Schweser et al., 2017). This means that finding an image support *S*_χ_ suitable for this incomplete spectrum approach is straightforward. Investigating the effect of eroding and dilating the mask in the numerical phantom showed that using a mask that was slightly larger than the brain region of interest does not negatively affect the QSM reconstruction (actually increasing PSNR in Figure 5). It should be noted, however, that a larger mask slows down the convergence of the method. The reconstruction converged more than twice as fast for 8 voxels of erosion with respect to the original mask: 7.3 seconds compared to 16.2 seconds with no erosion. The decrease in the XSIM metric compared to the PSNR metric as the mask is dilated highlights XSIM's increased sensitivity to local variance error (in this case outside of the original brain mask). Visually, neither the eroded nor the dilated reconstructions feature artifacts related to the choice of mask (see Figures 5C, D), which demonstrates the IS method to be relatively robust to masking (provided the background field removal was successful for the given mask).

Comparing the reconstructions in the numerical phantom (see Table 1 and Figure 6) we find that the incomplete spectrum performed slightly better than the direct TKD method but worse than the conventional state-of-the-art methods. Since the compressed sensing approach used such a small ℓ_1_ penalty term, adding the same regularization term to the incomplete spectrum reconstruction did not improve the metrics, although there is a visual difference between the regularized and unregularized IS reconstructions *in vivo* (see Figures 7A, B, 8). The ROI based analysis (Figure 6) shows a slight underestimation by the incomplete spectrum approach of the mean susceptibility in the globus pallidus (GP), caudate, red nucleus, thalamus and substantia nigra, compared to conventional methods except for the compressed sensing reconstruction which consistently underestimated the mean susceptibility in these ROIs. Overall, NDI performed the best on the challenge phantom as it provides ROI mean susceptibility values closest to the ground truth values except in the substantia nigra (Figure 4). This is not reflected in the XSIM and PSNR metrics (Table 1) nor in the *in-vivo* dataset ROIs (Figure 6), where NDI generally gave the lowest and least accurate reconstructed mean susceptibilities of the algorithms compared.

The “doubling” of the streaking artifact as observed, for example, around the calcification in the top of the brain in the simulated dataset, seems to be a side-effect of the way the incomplete spectrum method reconstructs the missing frequency domain information as illustrated in Figure 1. It is not as apparent *in vivo*, most likely because this dataset has a lower overall SNR. Since this IS method fills in k-space information between cones at two angles centered on the origin it replaces more high frequency than low frequency information, leading to a minor denoizing effect (observable as smoothing, specifically when comparing the *in vivo* TKD and incomplete spectrum reconstructions).

Since the incomplete spectrum method presented here does not rescale regions of the frequency domain (like TKD), it does not directly change the point spread function of the operator, and no rescaling of the output susceptibility map should be necessary [as was proposed for TKD by Schweser et al. (2013)]. This is supported by the incomplete spectrum results on the simulated dataset where no scaling in contrast was observed for higher levels of (intrinsic) regularization (although a slight loss in contrast due to smoothing was observed, as in the comparison between XSIM-optimal and PSNR-optimal reconstructions shown in Figures 3, 4).

### 5.2. *In vivo* reconstructions

Figure 10 shows that, *in vivo*, the incomplete spectrum approach gave ROI mean susceptibility values similar to those from commonly used, state-of-the-art algorithms i.e., NDI and nlTV. This suggests that the incomplete spectrum method did not systematically overestimate or underestimate the susceptibilities in these ROIs. In most of the ROIs, the incomplete spectrum approach gave susceptibility values between those from nlTV and TKD. The compressed sensing reconstruction gave the lowest susceptibility values in all ROIs. The literature values were larger than the values reconstructed by all algorithms in the caudate, putamen, substantia nigra (and globus pallidus) perhaps because the healthy volunteers were relatively young compared to the subjects included in the studies cited here and may, therefore, have had a lower iron content and a lower susceptibility in these deep-brain gray matter ROIs than the subjects in the cited studies (Li et al., 2014, 2023; Zhang et al., 2018).

With additional ℓ_1_ wavelet regularization, the incomplete spectrum QSM reconstruction more closely resembles the compressed sensing QSM (Wu et al., 2012) (Figures 7A–C, G–I, 8B, C, H, I). At high levels of regularization the regularized incomplete spectrum and compressed sensing reconstructions become identical, as expected from their similar cost functions (see Equations 12, 13), and illustrated in Supplementary Figure S1. Adding the regularization to the incomplete spectrum approach leads to less pronounced streaking (as illustrated by the orange arrow, Figures 7A, B). However, regularization adds another parameter to the method which then requires tuning, as opposed to the original implementation which is less sensitive to *t*_well_ initially tuned on the numerical phantom. Further, it has been shown that band-limiting based on thresholding the dipole kernel as performed in this incomplete spectrum approach leads to correlated artifacts (Wu et al., 2012) thereby violating the assumptions of compressed sensing (i.e. uncorrelated artifacts). Therefore, additional regularization using this compressed sensing term is disadvantageous compared to the original unregularized incomplete spectrum approach.

The biggest limitation of the incomplete spectrum QSM reconstruction approach lies in the input data ν = *D*^−1^*Fb*, which is the directly deconvolved phase information. Streaking artifacts are introduced by multiplication with the inverse dipole kernel *D*^−1^ and the incomplete spectrum method then “corrects” for these. Future work will involve applying the incomplete spectrum method to reconstructed susceptibility maps, to provide additional “correction” of the spectrum where applicable. This could lead to frequency domain correction schemes that would work in tandem with conventional iterative methods to improve the robustness to noise, or artifacts that can be identified in specific regions of frequency domain (such as streaking). This technique provides a new tool for filling in missing regions of frequency space, or correcting “ill-posed” or noisy frequency-space regions with a suitable band-limit in QSM maps by using an image space mask.

## Data availability statement

The data analyzed in this study is subject to the following licenses/restrictions: the anonymized *in-vivo* dataset and analysis will be provided by the authors upon reasonable request. Requests to access these datasets should be directed to KS, k.shmueli@ucl.ac.uk.

## Ethics statement

Ethical review and approval was not required for the study on human participants in accordance with the local legislation and institutional requirements. Written informed consent for participation was not required for this study in accordance with the national legislation and the institutional requirements.

## Author contributions

PF contributed to the conception and design of the study and performed the numerical analysis. KS was responsible for funding the research and gave feedback on the analysis. All authors contributed to manuscript writing and editing, as well as reading and approving the submitted version.
