# Supplementary material for: Incomplete spectrum QSM using support information
Source: Front Neurosci. 2023 Apr 17;17:1130524. doi: 10.3389/fnins.2023.1130524 (PMC10149841; doi:10.3389/fnins.2023.1130524)
Supplement: Supplementary file 1 [file Data_Sheet_1.PDF]

Supplementary Material

1 SUPPLEMENTARY TABLES AND FIGURES

1.1 Figures

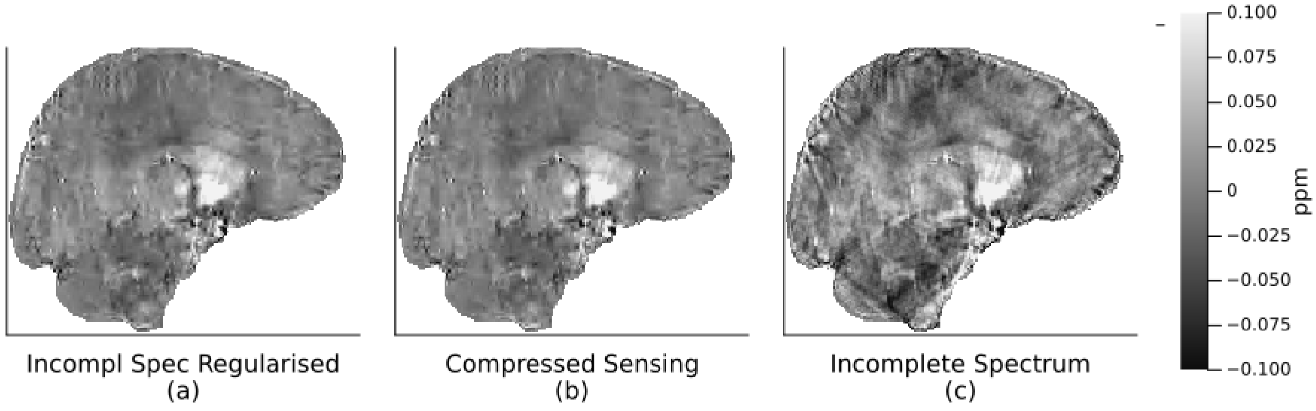

Figure S1. Reconstruction results for $\lambda_{CS} = 0.01$ regularisation weight (at a spectrum with threshold $t_{ill} = 0.28$).

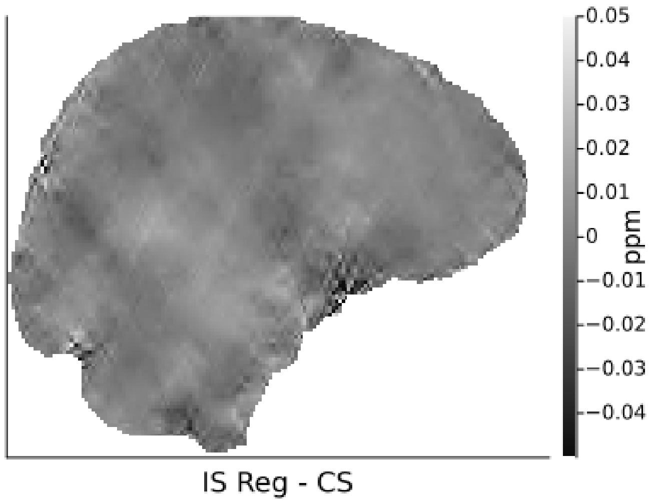

Figure S2. Difference between regularised incomplete spectrum and compressed sensing reconstructions at $\lambda_{CS} = 0.01$, not the scaled colorbar as compared to the differences presented in the paper.
